# Supplementary material for: Brain-actuated functional electrical stimulation elicits lasting arm motor recovery after stroke
Source: Nat Commun. 2018 Jun 20;9:2421. doi: 10.1038/s41467-018-04673-z (PMC6010454; doi:10.1038/s41467-018-04673-z)
Supplement: Supplementary file 1 — Supplementary Information [file 41467_2018_4673_MOESM1_ESM.pdf]

Brain-Actuated Functional Electrical Stimulation Elicits Lasting Arm Motor Recovery After Stroke

Biasiucci et al.

Supplementary Information

Supplementary Methods: BCI

During the calibration and therapeutic sessions, the EEG signal was recorded at 512 Hz sampling rate (g·tec gUSBamp, Guger Technologies OG, Graz, Austria), band-passed filtered within 0.1 and 100 Hz and notch filtered at 50 Hz. The monitored EEG channels were selected so as to adequately cover the sensorimotor cortex. Sixteen active surface electrodes were placed on positions Fz, FC3, FC1, FCz, FC2, FC4, C3, C1, Cz, C2, C4, CP3, CP1, CPz, CP2 and CP4 of the 10/20 International system. Reference was placed on right mastoid and ground on AFz. The signal was spatially filtered with a Laplacian derivation and the power spectral density (Welch periodogram) of each channel was computed with 2 Hz resolution in 1 s-long windows sliding every 62.5 ms. Feature selection was performed by ranking the candidate spatio-spectral features according to discriminant power, calculated through canonical variate analysis[1], eventually manually selecting the most discriminant and neurophysiologically relevant ones. A Gaussian classifier outputting a probability distribution over two MI tasks was used to classify the consecutive feature vectors in real time[2]. The Gaussian classifier was trained with a gradient-descent supervised learning approach (see below) using the dataset resulting from the calibration session. The samples with "uncertain" probability distributions (where the maximum probability does not exceed a certain threshold) were rejected, while the remaining ones were fed to an evidence accumulation module smoothing the classifier output by means of a leaky integrator (exponential smoothing). A final decision/FES trigger is emitted by the BCI system once the patient is able to push the integrated probabilities of the motor attempt class to reach the configurable confidence threshold. Upon delivery of FES, the integrated probabilities are reset to the uniform distribution so as to start an unbiased new trial.

We have purposefully not employed cross-validation. During the training phase of our Gaussian classifier we employed batch, iterative gradient-descent, as discussed by Leeb et al.[2]. There, we have opted for a 50/50 training/testing set split instead of cross-validation to reduce the training time. The estimated parameters of the gradient-descent iteration exhibiting the highest classification accuracy on the testing set were selected to build the final classifier using all the data in the calibration session, which was then employed throughout the therapy. On the other hand, all BCI accuracy results reported in the manuscript reflect the actual online accuracy

the subjects experienced during the therapy with the originally trained classification model, so that the use of cross-validation is inapplicable in this case.

Supplementary Methods: Functional electrical stimulation

The used commercial FES device (Krauth & Timmermann MotionStim8, Hamburg, Germany) had the following parameters: stimulation frequency (ranging from 16 to 30 Hz), current amplitude (ranging between 10 and 25 mA) and pulse width (500 μs), along with the shape/duration of the stimulation train and the electrode placement. Therapists configured free parameters at the beginning of each therapeutic session, before the first run, targeting the same movement across all patients and sessions. This strategy was based on the belief that, given the large variability of physiological responses and tolerance to the same FES train even in different sessions of the same participant, eliminating the FES as a confounding factor consists mostly in trying to trigger the same overt behavioral output (elicited movement) rather than balancing each individual parameter.

Available data demonstrated that the two free parameters (stimulation frequency and amplitude) did not confound our results. The stimulation frequency was in practice fixed to 25 Hz for all patients. This proved to be enough to achieve the desired movement for all patients. Regarding the stimulation amplitude for each single session, we are in possession of the data from 17/27 patients (8 BCI, 9 sham). The stimulation current was between 10 and 29 mA, with a total mean of 18.85 ± 4.78 mA. The maximum increase of simulation current between sessions 1 and 10 was +3 mA, while the biggest decrease was -5 mA. The mean difference across patients was only -0.53 mA. Most importantly, none of the following metrics exhibited significant differences between the two groups or correlated significantly with FMA improvement: i) average and standard deviation of current amplitude values used, ii) start values, iii) end values, iv) max-min value difference, v) maximum consecutive change, and vi) average value used. Only the maximum value used was significantly different between BCI and sham groups (two-tailed unpaired t-test, $p = 0.041$, average BCI: 18.4 mA, average sham: 23.0 mA). This effect is attributed to an "outlier" BCI participant, who was the only one to use the lowest possible stimulation current (10 mA). Even in this case the highest maximum amplitude was observed in the sham group, what intuitively should have promoted the sham rather than the BCI therapy.

Supplementary Methods: Functional connectivity/FMA correlations

We have performed additional analyses to strengthen our hypothesis that connectivity changes are correlated with improvement in the FMA scores. Particularly, we have analyzed the results from three perspectives: first, a model robustness analysis, where we perform a cross-validation on the goodness of the model; second, a confidence analysis, where we evaluate the confidence of the results obtained; and third, a predictive analysis, where we evaluate the predictive capabilities of our models again using cross-validation.

<u>Original analysis in the manuscript.</u> For the sake of comparison, we detail here the Pearson's correlation values between Δconnectivity and ΔFMA scores reported in the manuscript: $r(\mu) = 0.41$, $p = 0.045$; $r(\beta) = 0.48$, $p = 0.02$.

<u>Model robustness evaluation.</u> We evaluated the Pearson's correlations obtained using cross-validation. Due to the low number of examples, we chose leave-one-out cross validation, where each fold is composed of all the samples but one, thus leading to as many folds as samples. The correlations obtained with this evaluation were (MEAN±SEM): $r(\mu) = 0.42 \pm 0.006$ (12 out of 24 correlations are significant ($p < 0.05$); minimum $p = 0.01$, maximum $p = 0.08$); $r(\beta) = 0.48 \pm 0.007$ (23 out of 24 correlations are significant ($p < 0.05$); minimum $p = 0.005$, maximum $p = 0.06$). This result substantiates that the correlation was not driven by single outliers in the data.

<u>Confidence analysis.</u> We assessed the confidence of the obtained correlation values using bootstrapping with replacement. Bootstrapping is a statistical resampling technique that extracts a measure of confidence/accuracy on the metric of interest. In our case, the metric of interest is the correlation between Δconnectivity and ΔFMA. The outcome of this bootstrapping is a confidence interval of correlation values, which can be interpreted as a correlation significantly different from zero if the confidence interval does not include the zero. We performed 5000 iterations of bootstrapping with replacement to build athe distribution of correlation values, and extracted the confidence values from such distributions (**Supplementary Fig. 6**). The confidence intervals at 95% for both correlations were of $r(\mu) \in [0.13, 0.64]$ and $r(\beta) \in [0.07, 0.70]$. Thus, we concluded that our correlations were significantly different from zero with 95% confidence.

<u>Predictive analysis.</u> To strengthen the validity of the results obtained, we built linear models to assess the predictive capabilities ΔFMA scores (dependent variable) from Δconnectivity (independent variable). Similarly to the FMA-accuracy model (see manuscript), we built

regressors using leave-one-out cross validation that were tested on the remaining testing example, and evaluated using r^2. With this approach, the results obtained were of $r^2(\mu) = 0.24$; $r^2(\beta) = 0.26$, and thus functional connectivity of alpha and beta bands alone explained 25% of the total variance of the FMA scores. Interestingly, a linear model considering both alpha and beta connectivity frequency bands boosted even more the variance explained, $r^2(\mu, \beta) = 0.36$.

Supplementary Methods: Neural desynchronization and source localization

We performed source localization from the surface EEG to determine the differences in activation at the voxel level following the therapy. We used standardized low-resolution brain electromagnetic tomography (sLoreta)[3], calculating the current density of each voxel and projecting it onto the Montreal Neurological Institute (MNI) standard brain.

We measured the task-related neural oscillatory changes at the voxel level and compared them between the two groups. To do so, we estimated the inverse solution with the 64-channel filtered EEG. From these data and for each subject, we computed the averaged cross-spectrum for the two conditions (movement attempt and rest) at the frequencies of interest. Then, we calculated the relative neural oscillatory desynchronization/synchronization by subtracting rest from movement attempt voxel activation.

From the results obtained we performed a non-parametric statistical test at the group level (BCI vs sham), after the intervention. The test was based on estimating, via randomization (5000 permutations), the empirical probability distribution for the max t-statistic under the null hypothesis, and automatically correcting for multiple comparisons. The output of the test was the voxels with significant differences ($p < 0.05$ corrected from the sampled t-distributions) between the two groups, together with the MNI coordinates and Brodmann areas involved.

After computing the inverse solution sLoreta, we found a significant change of neural desynchronization during the motor task at the voxel level between groups after the intervention for μ (non-parametric corrected permutation test, minimum $p = 0.0002$) but not for β although the desynchronization was also larger on average (minimum $p = 0.18$) (**Supplementary Fig. 7**). For both frequency bands, maximum t-value was found at the same MNI coordinates (μ: value = -8.86; β: value = -1.06; $X = 5$, $Y = -65$, $Z = 65$), with best matches in Brodmann Areas 7, 5, 4 and 3 (somatosensory and motor cortices). These changes were present in the affected and

healthy hemispheres. No significant differences in somatosensory and motor cortices were found between the two groups prior to the intervention.

In order to assess the robustness of the results at the source space, we performed the same tests using leave-one-subject-out cross-validation. For all the folds except for one, maximum t-value was found at the same MNI coordinates, and the results were significant for 9/12 (75%) of the folds.

In summary, neural desynchronization at μ and β frequency bands in the regions of interest were larger (significant and robust across subjects in the case of μ) for the BCI group compared to the sham group after intervention.

Supplementary Methods: Investigation on influence of artifacts

Given the importance of contingency between motor-related cortical signals and FES delivery, it is necessary to show that BCI performance was not affected by artifacts so that motor decoding inferences were actually corresponding to physiological motor EEG correlates.

First, we prove that any influence of facial EMG on the raw EEG signal during motor attempt was minimal. Specifically, for each therapeutic run, the statistics (average, standard deviation) of the artifact-free EEG signal of all channels is estimated after low-pass filtering with cutoff at 20 Hz, given that facial EMG are known to occur above this frequency[4],[5]. Subsequently, we measure the percentage of time that the original, non-filtered (and, potentially, artifact-contaminated) signal is above a certain z-score threshold computed on the artifact-free signal. Values above a z-score of 3.0 are conventionally thought to represent abnormally high amplitudes, indicative of EMG artifacts. This analysis revealed that during motor-attempt 3.03% of the EEG samples had an absolute z-score above threshold.

Second, in order to quantify how our BCI was driven by the desired SMR features and not by task-specific artifacts, all 14 patients in the BCI group exhibited μ and/or β sensorimotor rhythms as control signals, what suggests the BCIs built for the therapy do not rely on artifacts. This was derived from the average (across all runs/sessions) feature discriminability between the PSD samples of motor attempt and rest (**Supplementary Fig. 1**).

Third, BCI accuracy of subjects in the BCI group highly correlated with the discriminancy (average Fisher score) of the selected SMR features (Pearson's correlation, $r = 0.57$, $p = 0.002$). This is not true for the average discriminancy of spectral features of the Fz channel (Pearson's

correlation, $r = 0.24$, $p = 0.223$), which is the channel most susceptible to facial and ocular artifacts.

Supplementary References

[1] Galán F, Ferrez PW, Oliva F, Guàrdia J, Millán JdR. Feature extraction for multi-class BCI using canonical variates analysis. In *IEEE Int Symp Intell Signal Proc* (2007).

[2] Leeb R, Perdikis S, Tonin L, Biasiucci A, Tavella M, Creatura M, et al. Transferring brain-computer interfaces beyond the laboratory: successful application control for motor-disabled users. *Artif Intell Med* **59**, 121–132 (2013).

[3] Pascual-Marqui RD. Standardized low-resolution brain electromagnetic tomography (sLORETA): technical details. *Methods Find Exp Clin Pharmacol* **24**, 5–12 (2002).

[4] Goncharova II, McFarland DJ, Vaughan TM, Wolpaw JR. EMG contamination of EEG: spectral and topographical characteristics. *Clin Neurophysiol* **114**, 1580–1593 (2003).

[5] Van Boxtel A. Facial EMG as a tool for inferring affective states. In *Measuring Behaviour Conf*, 104–108 (2010).

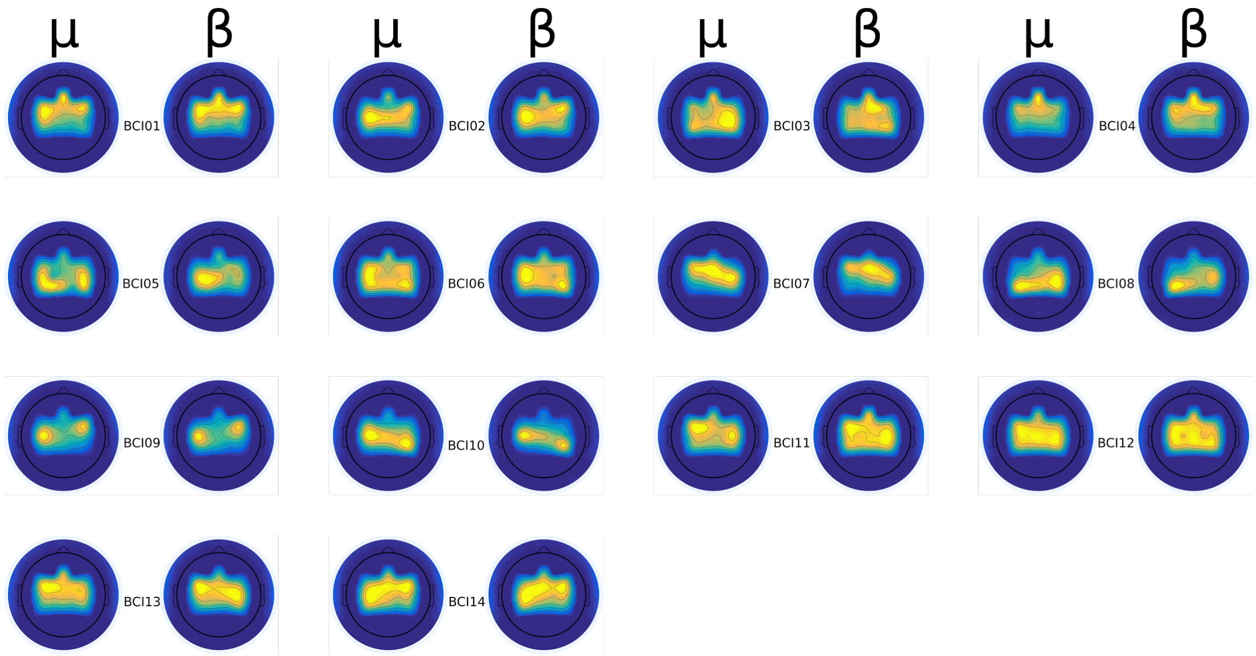

Supplementary Figure 1: Sensorimotor rhythm modulation in online therapeutic sessions. Sensorimotor rhythm patterns in the mu (8-14 Hz) and beta (18-24 Hz) bands of patients in the BCI group during the therapeutic sessions. Yellow corresponds to the maximum discriminancy (Fisher Score of the corresponding PSD values between "rest" and "motor attempt") value within each map. The affected hemisphere is on the left.

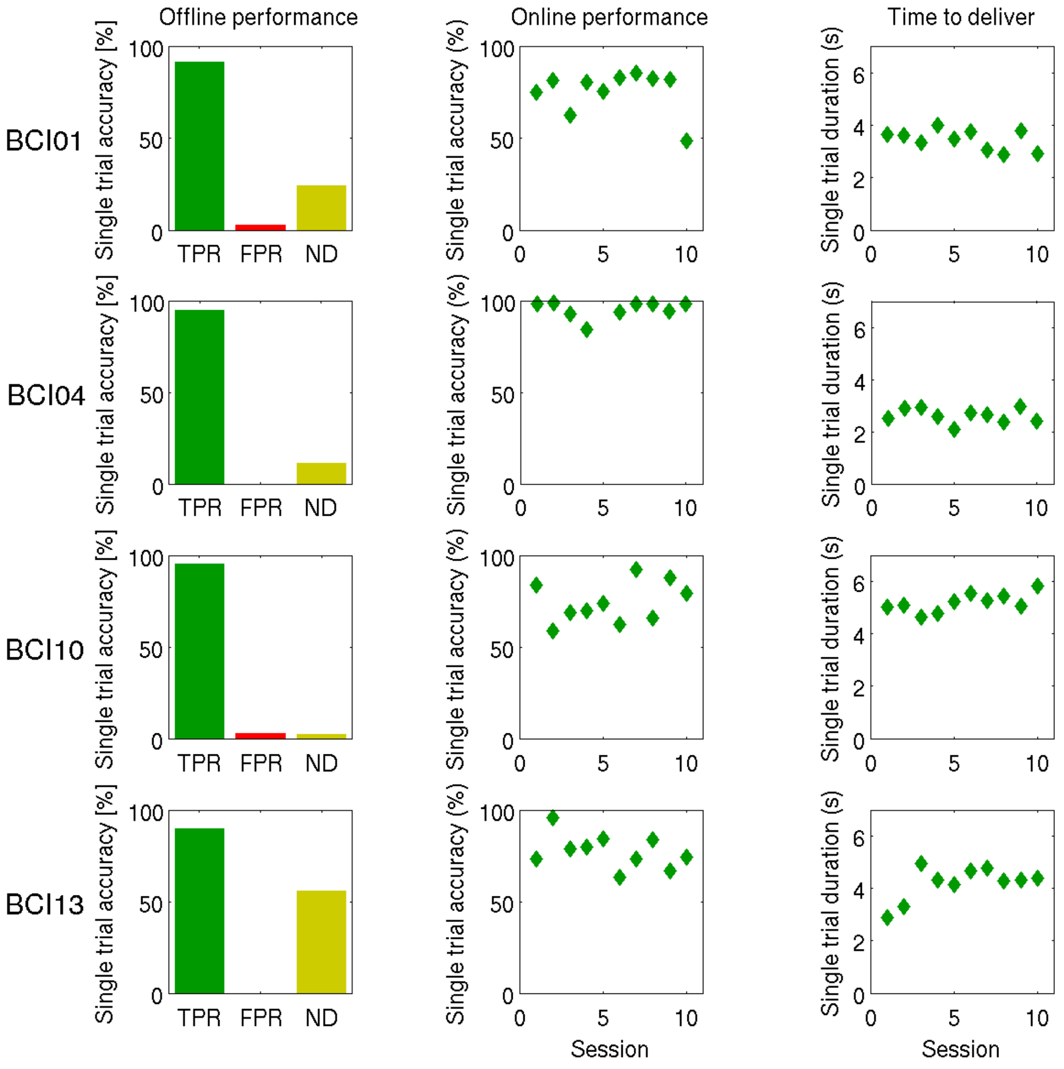

Supplementary Figure 2: BCI performance of four exemplary patients illustrating the range of results. *Left.* Offline single-trial performance estimated in the calibration: true positive rate (TPR), false positive rate (FPR) and no-decision (ND). *Center.* Online single-trial classification performance for each session. *Right.* Average time required by the BCI to detect a movement attempt in each session.

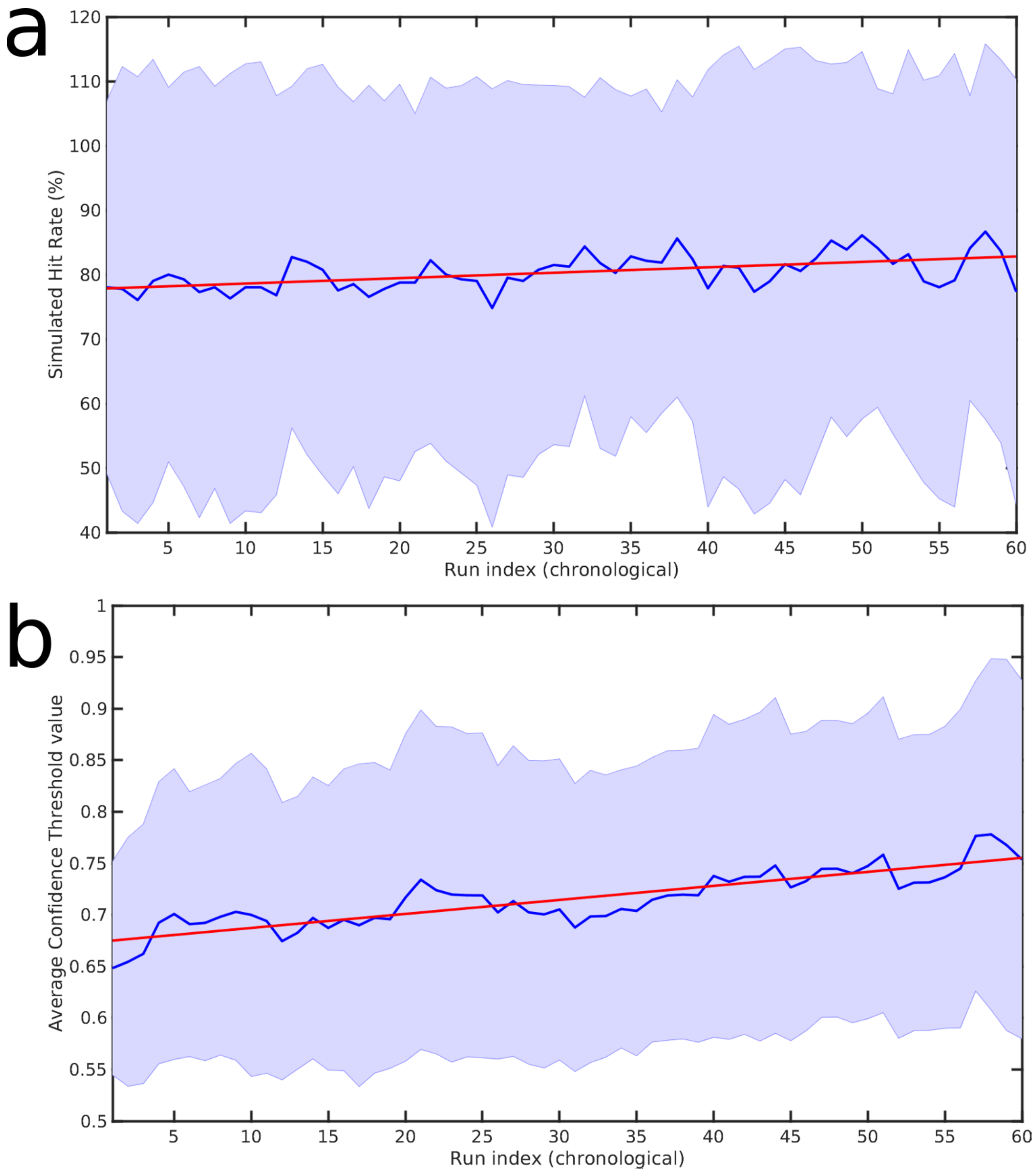

Supplementary Figure 3: Evolution of simulated hit rate and confidence threshold throughout the intervention. Grand average (blue lines), standard deviation (shadows), and corresponding linear fits (red lines) across BCI group participants of a) simulated hit rate over runs with a conservative fixed threshold, and b) confidence threshold value.

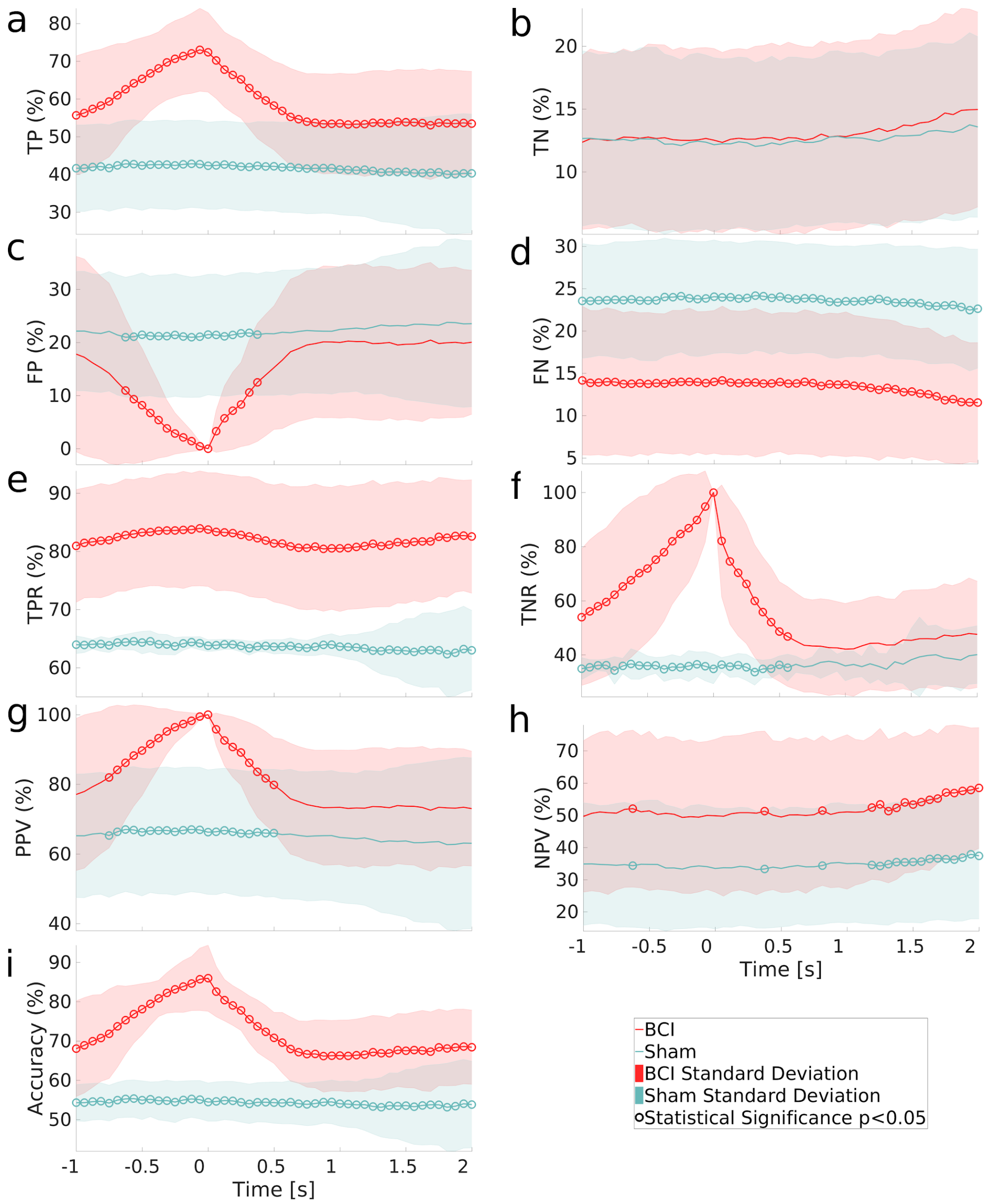

Supplementary Figure 4: Contingency metrics between SMR detection and FES over sliding PSD windows in the interval [-1, 2] s surrounding the end of motor attempt trials (FES onset for "hit trials"). Mean and standard deviation are plotted for BCI and Sham groups for metrics: (a) TP, (b) TN, (c) FP, (d) FN, (e) TPR, (f) TNR, (g) PPV, (h) NPV, (i) Accuracy. Circular dots denote significant ($p<0.05$) group (BCI vs Sham) differences for the respective metric at that time point. The time axis refers to the position of the right-side edge of the 1-sec long sliding PSD windows, so that t=0 is the PSD window at the FES onset.

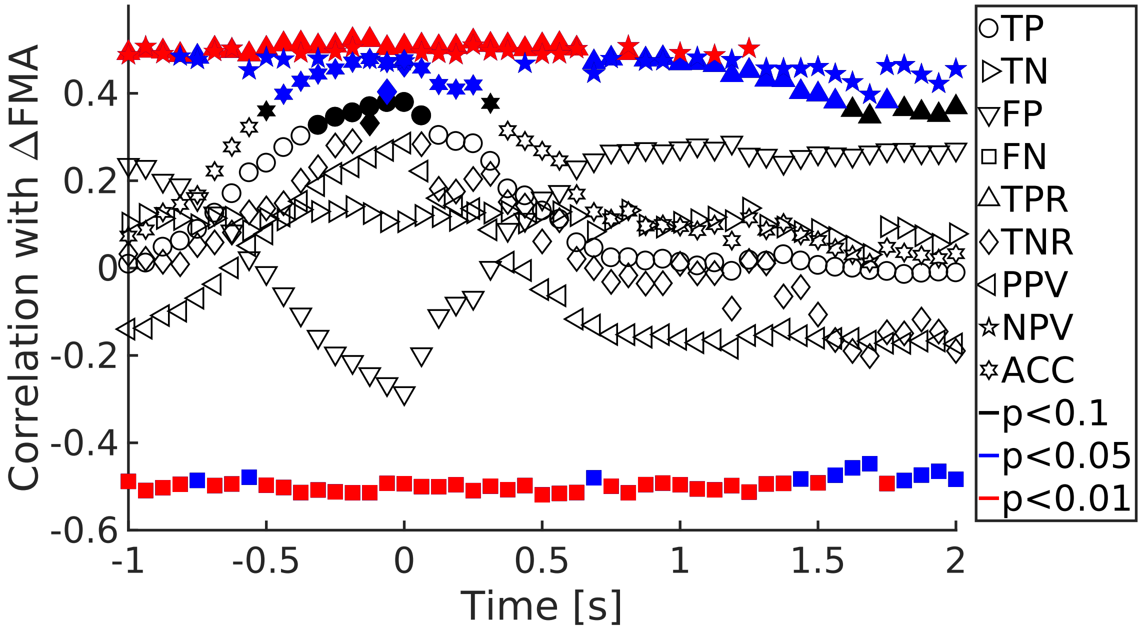

Supplementary Figure 5: Pearson correlations of motor recovery (ΔFMA) with contingency metrics over sliding PSD windows in the interval [-1, 2] s surrounding the end of motor attempt trials (FES onset for "hit trials"). Metric type and statistical significance (most conservative confidence interval) as denoted in the legend. The time axis refers to the position of the right-side edges of the 1-sec long sliding PSD windows, so that t=0 is the PSD window at the FES onset. Filled colored bold icons indicate the significance level (black p <0.1, blue p <0.05, red p <0.01, blank p >0.1) for each of the nine metrics.

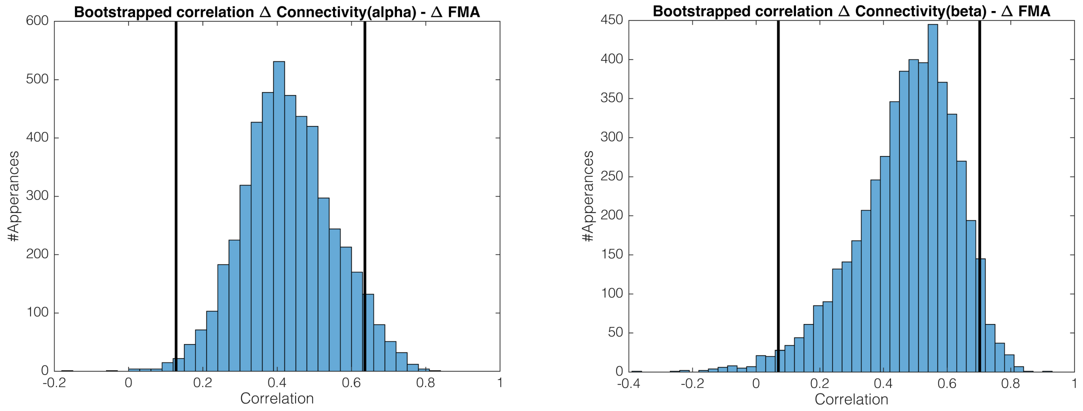

Supplementary Figure 6: Correlation bootstrapping distributions (histogram, based on 5000 permutations with replacement) for the mu (left) and beta (right) frequency bands. Black thick lines represent the lower and upper confidence bounds at 95%.

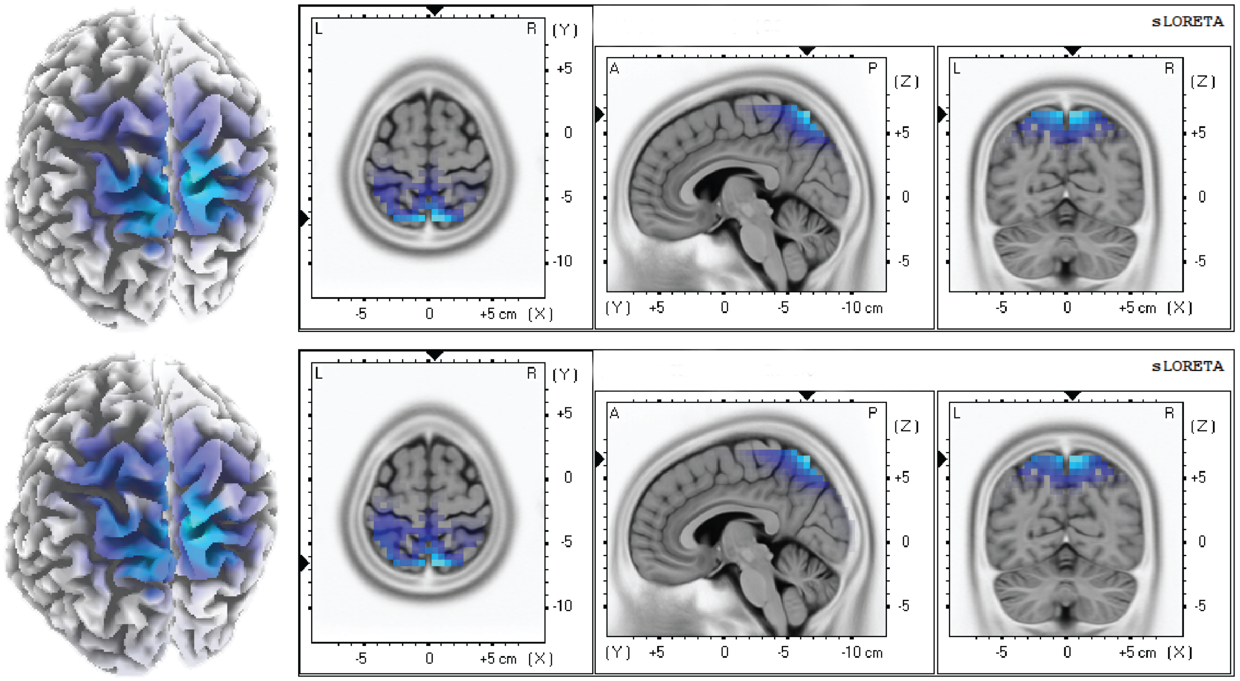

Supplementary Figure 7: sLoreta source localization results during movement attempts. BCI vs sham neural desynchronization at the voxel level (blue: larger desynchronization for the BCI group) after intervention for μ (10-12 Hz, top) and β (18-24 Hz, bottom) frequency bands. Significant voxel activations were only found for the μ band. Electrodes were flipped so the lesion was always located on the left.

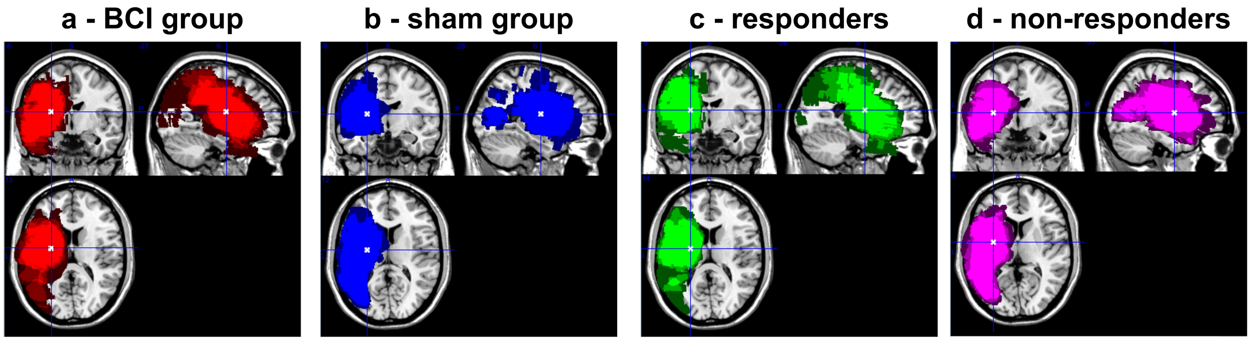

Supplementary Figure 8: Average center of gravity and distribution of lesions. Average center of gravity marked with crosshair and average distribution of lesions for *(a)* BCI group, *(b)* sham group, *(c)* responders (ΔFMA > 4), and *(d)* non-responders (ΔFMA ≤ 4). Right hemisphere lesions are mirrored to the left side. No significant differences between the groups have been found neither for lesion volume nor for center of gravity.

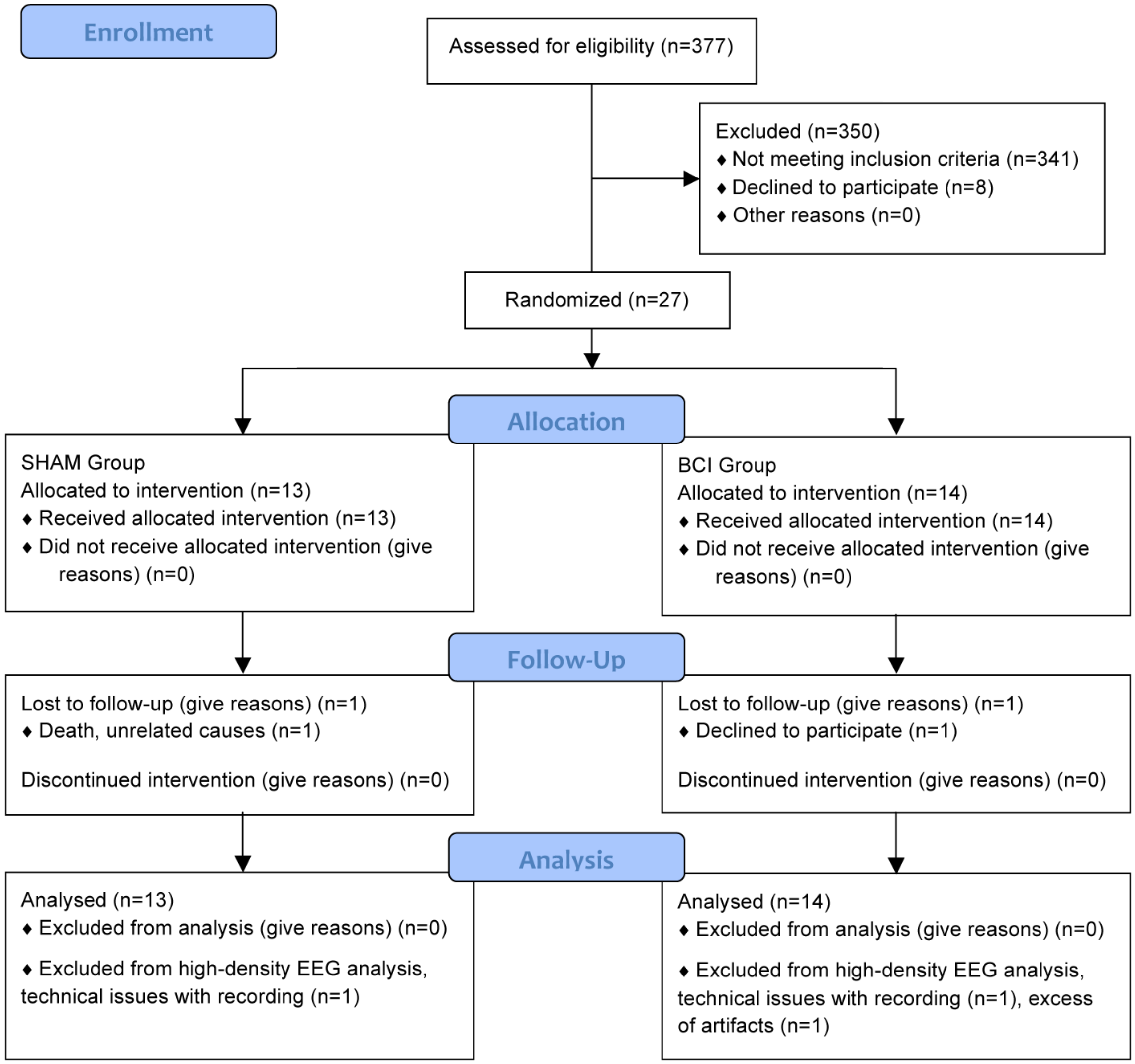

Supplementary Figure 9: CONSORT flow diagram. Study enrollment diagram. 377 patients were screened and 27 were eligible and agreed to participate. Patients were recruited sequentially and randomly assigned to 2 groups: 14 participants in the BCI-FES group and 13 participants in the sham-FES group. In the BCI group, detection of an appropriate pattern of brain activity activated FES of the extensor digitorum communis muscle. In the sham group, FES of the same muscle was delivered randomly. Two patients, one per group, could not do the follow-up clinical evaluation.

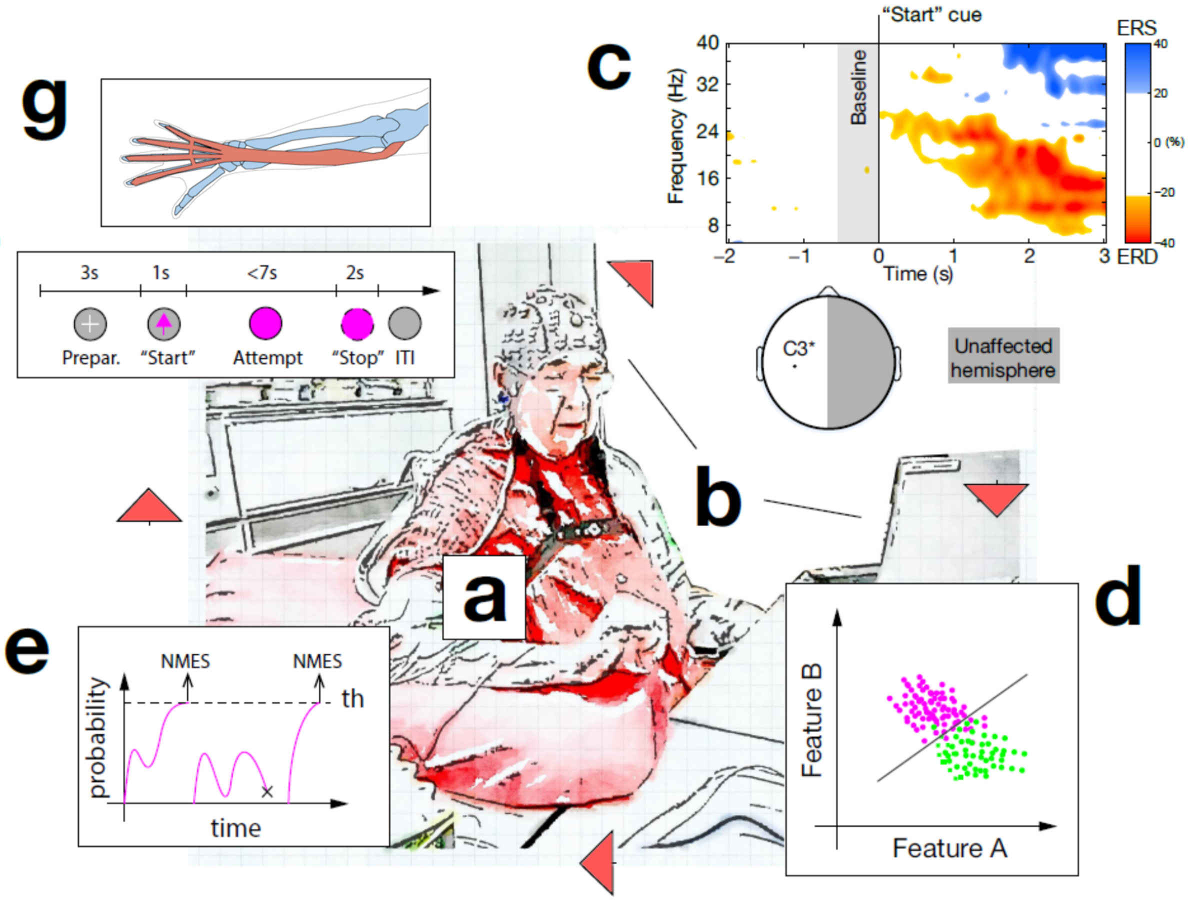

Supplementary Figure 10: Brain-actuated functional electrical stimulation. *(a)* During the therapy, participants sat comfortably and were asked to concentrate on their affected limb for the whole session (Credit: Dr. Andrea Biasiucci). *(b)* For the BCI-actuated functional electrical stimulation group (BCI-FES), a BCI was individually calibrated to distinguish hand-extension attempts from resting. Data to build the BCI classifier were acquired during a calibration session before starting the therapy. *(c)* Selected discriminant EEG features were localized in the ipsi- and contra-lesional motor areas in frequency bands normally associated with voluntary movements (i.e., in the μ and β bands). *(d)* These subject-specific features were the input for a statistical classifier that responded with the probability distribution that the current EEG feature vector belongs to each of the two classes (movement attempt or resting). *(e)* The statistical classifier computed probabilities from EEG feature vectors 16 times per second, and the BCI accumulated them until a confidence threshold was reached. *(f)* A trial started with the "Preparation" cue (a cross in the middle of the screen) during 3 s, then a "Start" cue appeared for 1 s indicating that the subject had to attempt extension of affected hand, which was sustained for up to 7 s (trial

time-out), and finished with the appearance of the “Stop” cue during 2 s. Inter-trial intervals lasted from 3 to 4.5 s. *(g)* Whenever a hand-extension attempt was decoded during the 7-s period, the BCI activated FES of the *extensor digitorum communis* muscle (Credit: Dr. Andrea Biasiucci). Patients in the sham-FES group wore identical hardware and received identical instructions as BCI-FES patients, but FES was not driven by neural activity and it was instead delivered randomly with similar timing and amount of stimulation. Both therapies lasted 10 sessions.

Supplementary Table 1. Contingency table between motor attempt detection and FES.

		**Motor Attempt Detection**	
		Yes	*No*
**FES Stimulation**	*Yes*	True Positives (TP)	False Positives (FP)
	No	False Negatives (FN)	True Negatives (TN)
